# 双抑制在线基体去除-离子色谱法测定电池级碳酸锂中痕量阴离子

**DOI:** 10.3724/SP.J.1123.2023.10003

**Published:** 2024-03-08

**Authors:** Gang WU, Guoquan WU

**Affiliations:** 1.正大天晴药业集团股份有限公司, 江苏 连云港 222062; 1. Chia Tai Tianqing Pharmaceutical Group Co., Ltd., Lianyungang 222062, China; 2.赛默飞世尔科技(中国)有限公司广州应用研发中心, 广东 广州 510800; 2. Guangzhou Application R& D Center, Thermo Fisher Technology (China) Co., Ltd., Guangzhou 510800, China

**Keywords:** 离子色谱, 阴离子, 碳酸锂, 在线基体去除, 双抑制, ion chromatography (IC), anions, lithium carbonate, on-line matrix-removal, double-inhibition

## Abstract

建立了一种测定电池级碳酸锂中痕量阴离子的方法。用超纯水超声辅助溶解碳酸锂,采用在线基体去除法去除碳酸锂基体,基体去除过程中,样品首先流入到ADRS600(4 mm)抑制器(抑制电流150 mA,抑制器再生液外接水流速2 mL/min)中,在抑制器内通过离子交换膜作用,碳酸锂中的锂离子与氢离子置换,碳酸锂变成碳酸;然后经过CRD 200(4 mm),碳酸以二氧化碳的形式排出离子色谱系统,从而达到去除碳酸锂基体的目的;最后待分析阴离子被自动富集在IonPac UTAC-LP2浓缩柱(35 mm×3 mm)上,通过阀切换技术将阴离子自动转移到色谱分析系统中。在色谱分析系统中,以IonPac AG18(50 mm×2 mm)为保护柱,IonPac AS18(250 mm×2 mm)为分析柱,柱温箱为30 ℃, KOH溶液为淋洗液进行梯度洗脱,泵流速0.30 mL/min,抑制器为ADRS600(2 mm),抑制电流25 mA,进样体积为250 μL,检测器为电导检测器。结果显示:F^-^、Cl^-^、NO_2_^-^、Br^-^、NO_3_^-^及SO_4_^2-^离子在各自的线性范围内具有良好的线性关系(r≥0.999);各离子的检出限和定量限分别为0.05~0.88 μg/L和0.15~2.92 μg/L;碳酸锂样品连续6针进样各离子峰面积的相对标准偏差(RSD)均≤0.73%;同一碳酸锂样品处理完后分别放置0、2、4、8、12、18、24 h后进样,各离子峰面积的RSD均≤0.96%;在3个添加水平下,各离子加标回收率为93.3%~99.3%, RSD(n=6)为0.97%~3.45%;本方法具有方法定量限低(各离子定量限均为0.5 mg/kg)及多种离子同时分析的优势,适用于电池级碳酸锂中痕量阴离子的检测。

## Abstract (en)

A method was developed for the determination of trace anions in battery-grade lithium carbonate. In this method, lithium carbonate was dissolved in ultrapure water with ultrasound assistance, and its matrix was removed using an on-line matrix-removal method. In the matrix-removal process, the sample was first passed through an ADRS600(4 mm) suppressor (suppressor current, 150 mA; external water flow rate, 2 mL/min). Hydrogen and lithium ions were then completely exchanged via the ion-exchange membrane in the suppressor, converting the lithium carbonate into carbonic acid. The carbonic acid entered the waste-liquid channel in the form of carbon dioxide through a CRD 200(4 mm) carbonate removal device to remove the lithium carbonate matrix. Finally, the target anions were automatically enriched on an IonPac UTAC-LP2 concentration column (35 mm×3 mm) and automatically transferred to a chromatographic system using valve-switching technology. The chromatographic system featured an IonPac AG18 column (50 mm×2 mm) as the protection column and an IonPac AS18 column (250 mm×2 mm) as the analytical column. The column temperature was 30 ℃, gradient elution was performed using KOH solution as the eluent, and the pump flow rate was 0.30 mL/min. An ADRS600(2 mm) suppressor, suppressor current of 25 mA, injection volume of 250 μL, and conductance detector were also used. The results showed good linear relationships (*r*≥ 0.999) for F^-^, Cl^-^, NO_2_^-^, Br^-^, NO_3_^-^, and SO_4_^2-^ in their respective concentration ranges. The limits of detection (LODs) and quantification (LOQs) were 0.05-0.88 and 0.15-2.92 μg/L, respectively. Lithium carbonate samples were tested six consecutive times, and the relative standard deviations (RSDs) of the peak areas of each ion were less than 0.73%. The same lithium carbonate samples were injected after 0, 2, 4, 8, 12, 18, and 24 h, and the RSD of the peak areas of each ion was less than 0.96%. The average recoveries ranged from 93.3% to 99.3%, and the RSDs (*n*=6) of samples spiked at three levels were in the range of 0.97%-3.45%. The proposed method has a low method limit of quantification of only 0.5 mg/kg for each ion analyzed and is capable of the simultaneous analysis of multiple ions. Thus, it is suitable for the detection of trace anions in battery-grade lithium carbonate.

锂离子电池(LIBs)作为化学能和电能相互转换的装置,广泛应用于便携式电子设备、新能源汽车、军事航天等领域^[1,2]^。高纯碳酸锂产品包括主含量99.9%、99.99%、99.999% 3种,其中99.99%主含量的高纯碳酸锂产品在市场上应用最为广泛^[3]^。国内生产企业主要采用的生产工艺为以精制氢氧化锂为原料,采用碳酸盐或二氧化碳沉淀法生产高纯碳酸锂;或者以碳酸锂为原料,采用氢化后热分解工艺生产高纯碳酸锂^[4]^。

针对碳酸锂,我国的标准主要有GB/T 11075-2013《碳酸锂》和YS/T 582-2013《电池级碳酸锂》^[5,6]^,标准中规定了电池级碳酸锂中Cl^-^及 ${{\mathrm{SO}}_{4}}^{2-}$ 的限量分别为≤0.003%和≤0.08%,推荐检测方法如下:Cl^-^与银离子生成氯化银沉淀, ${{\mathrm{SO}}_{4}}^{2-}$ 与钡离子生成硫酸钡沉淀,分别用分光光度计测其吸光度计算Cl^-^和 ${{\mathrm{SO}}_{4}}^{2-}$ 含量,以上检测方法仪器设备投入少,成本低,但容易受到基体干扰,检测结果偏差较大。离子色谱是常规强极性小分子离子分析的首选方法,采用离子色谱检测碳酸锂中的杂质离子也有相关报道^[7⇓⇓-10]^,文献[7⇓-9]均是采用离线On Guard H柱消除碳酸根干扰,采用该方式只能将部分碳酸根转化成碳酸氢根,无法去除,大体积进样时仍会对F^-^、Cl^-^产生干扰,不适用于痕量离子分析,无法兼容大体积进样,实现痕量离子的检测;同时部分方法中采用酸溶解样品,容易引入污染,影响检测结果;文献[10]采用在线基体去除-离子色谱法检测碳酸锂中Cl^-^及 ${{\mathrm{SO}}_{4}}^{2-}$ ,但去除装置为抑制柱和二氧化碳抑制器(MCS),由于抑制柱通过离子交换去除锂离子,无法实现连续性,故有限容量无法兼容大体积及高基体样本,无法兼容痕量离子分析;亦有报道采用电感耦合等离子体质谱(ICP-MS)检测碳酸锂中的SO_4_^2-^^[11]^, ICP-MS法具有无基体干扰优势,但样品需要用酸溶解,容易引入污染,且无法同时兼容样品中F^-^、Cl^-^、 ${{\mathrm{NO}}_{2}}^{-}$ 、 ${{\mathrm{NO}}_{3}}^{-}$ 、Br^-^及 ${{\mathrm{SO}}_{4}}^{2-}$ 的检测,应用范围受限。

本方法对文献[7⇓⇓-10]的检测方法进行改进,采用在线自动去除碳酸锂基体,实现了目标离子浓缩,兼容大体积进样,满足了超低痕量离子的检测需求;采用超纯水溶解样品,无杂质离子引入,可兼容6种阴离子的同时分析。

## 1 实验部分

### 1.1 仪器、试剂与材料

Dionex ICS-6000双系统离子色谱仪(含双泵及双进样阀)、AXP泵、FB15065超声波分析仪器、ADRS600(4 mm)及ADRS600(2 mm)阴离子动态再生抑制器、CRD 200(4 mm)二氧化碳去除器,均为美国Thermo Fisher公司产品。XPE205DR型十万分之一天平(美国Mettler Toledo公司), Milli-Q型超纯水仪(德国Millipore公司)。

F^-^、Cl^-^、 ${{\mathrm{NO}}_{2}}^{-}$ 、Br^-^、 ${{\mathrm{NO}}_{3}}^{-}$ 和 ${{\mathrm{SO}}_{4}}^{2-}$ 标准溶液(质量浓度均为1000 mg/L),购自国家标准物质研究中心。电池级碳酸锂样品购自市场。

### 1.2 标准溶液的配制

分别准确移取Cl^-^、 ${{\mathrm{NO}}_{2}}^{-}$ 、Br^-^、 ${{\mathrm{NO}}_{3}}^{-}$ 标准溶液1.0 mL于100 mL容量瓶中,加超纯水稀释至刻度,配制成混合标准溶液1;移取10.0 mL F^-^及 ${{\mathrm{SO}}_{4}}^{2-}$ 标准溶液于100 mL容量瓶中,加超纯水稀释至刻度,配制成混合标准溶液2;分别取适量混合标准溶液1及2,配制系列标准溶液,其中Cl^-^、 ${{\mathrm{NO}}_{2}}^{-}$ 、Br^-^、 ${{\mathrm{NO}}_{3}}^{-}$ 质量浓度为5.0、10.0、20、40、80、100及200 μg/L,F^-^及 ${{\mathrm{SO}}_{4}}^{2-}$ 质量浓度为5.0、50.0、100、200、500、2000、4000 μg/L,待用。

### 1.3 样品的制备

精密称取0.50 g碳酸锂样品,置于50 mL容量瓶中,用超纯水定容,于50 ℃超声(功率380 W,频率37 kHz)溶解15 min,摇匀,得到1.0%(质量分数)的样品溶液,过0.45 μm水相滤膜后进样。

### 1.4 色谱条件

保护柱为IonPac AG18 (50 mm×2 mm),分析柱为IonPac AS18 (250 mm×2 mm),柱温为30 ℃,淋洗液为KOH,梯度洗脱(0~12 min,淋洗液浓度为5 mmol/L;12~25 min,淋洗液浓度为5~25 mmol/L;25~30 min,淋洗液浓度为25 mmol/L);进样量为250 μL,泵1流速为0.25 mL/min,泵2流速为0.30 mL/min, AXP泵流速为2.0 mL/min, ADRS600(4 mm)抑制器电流为150 mA; ADRS600(2 mm)抑制器电流为25 mA。

## 2 结果与讨论

### 2.1 样品前处理条件的优化

碳酸锂在酸性条件下易于溶解,本方法考察了以0.1%甲基磺酸、0.1%高氯酸及纯水分别作为溶剂溶解碳酸锂的效果,结果表明,在加热条件下0.1%甲基磺酸及0.1%高氯酸均可快速溶解碳酸锂,但分别存在以下问题:甲基磺酸具有电导响应,0.1%的甲基磺酸响应峰对F^-^、Cl^-^及 ${{\mathrm{NO}}_{2}}^{-}$ 干扰严重,无法满足痕量离子的检测要求;高氯酸根具有强保留特性,其保留能力强于 ${{\mathrm{SO}}_{4}}^{2-}$ ,但其具有强氧化性,容易引入Cl^-^导致无法准确定量;样品采用酸溶方式,虽可快速溶解样品,但受基体效应影响,无法兼容大体积进样,满足超低痕量分析。超声是一种简便高效的溶解方法,实验证明0.5 g电池级碳酸锂在超声条件下可完全溶于50 mL超纯水中,且溶解过程中无任何杂质离子引入。与酸溶方法相比,虽然采用超纯水溶解样品稀释倍数相对较大,但本方法可通过加大进样量来提高分析灵敏度,综合考虑,本方法最终选择超纯水溶解碳酸锂。

### 2.2 色谱条件优化

#### 2.2.1 基体去除条件确定

碳酸锂水溶后含有大量的碳酸根,高浓度的碳酸根会发生自淋洗,影响基线,无法准确定量痕量杂质离子。本方法对比了3种降低基体效应的方式:①将1.0%碳酸锂样品溶液过离线On Guard H柱中和碳酸根;②将1.0%碳酸锂样品溶液通过在线In Guard H柱中和碳酸根; ③将1.0%碳酸锂样品溶液经过ADRS600(4 mm)抑制器及CRD 200(4 mm)将碳酸锂基体在线去除(见图1)。结果表明,采用On Guard H柱进样体积受限,手动操作容易引入杂质离子干扰,不适用于痕量离子的检测;采用In Guard H柱方法优于On Guard H柱方法,但进样体积仍受限,原因为高含量的碳酸根经过In Guard H柱后会产生大量的碳酸氢根,高含量的碳酸氢根影响基线,弱保留离子无法准确定量。采用ADRS600(4 mm)抑制器及CRD 200(4 mm)在线去除碳酸锂基体,过程如下:借助泵的动力,样品首先经过ADRS600(4 mm)抑制器,在抑制器内通过离子交换膜,氢离子与锂离子完成再生液通道与淋洗液通道的置换,锂离子被排入抑制器再生液通道(即废液通道),同时碳酸锂在抑制器淋洗液通道内转变成碳酸;然后经过CRD 200(4 mm),碳酸以二氧化碳的形式从CRD 200的废液通道排出,从而达到去除碳酸锂基体的目的;最后待分析杂质阴离子被自动富集在IonPac UTAC-LP2浓缩柱(35 mm×3 mm,美国Thermo Fisher公司)上,通过阀切换技术将待分析离子自动转移到色谱分析系统中,整个过程目标杂质离子无任何损失且被浓缩在浓缩柱中,无任何基体及外界离子进入到离子色谱分析系统中,因此该模式可有效去除基体干扰,兼容大体积进样。经测试,采用本方法进样体积增至1 mL时不会对基体产生任何影响,本方法考虑常规环境下检出限要求,将进样体积定为250 μL。

**图 1:**
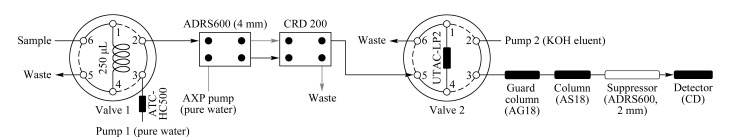
在线连续去除碳酸锂基体系统连接图

#### 2.2.2 泵1流速、抑制电流及外接水流速确定

由图1系统连接图可知,泵1流速影响样品前处理时间,ADRS600(4 mm)抑制器电流影响抑制效果(即碳酸锂转化为碳酸的效率),理论上泵1流速越低及ADRS600(4 mm)抑制器电流越大效果越好,但考虑到分析效率及抑制器的最佳工作状态,将泵1流速设置为0.25 mL/min,并以5倍定量环体积冲洗定量环,在此条件下,5 min(共1.25 mL液体量)即可满足基体在线去除、杂质阴离子自动富集在浓缩柱上的要求。经实验证实,结合泵1 0.25 mL/min的流速,将抑制器电流设置为150 mA时足以将碳酸锂中的锂离子全部去除,故将抑制器电流设置为150 mA。AXP泵为ADRS600(4 mm)抑制器再生液通道提供外接水,结合抑制器电流情况,将AXP泵流速设置为2.0 mL/min。

#### 2.2.3 色谱柱选择

本方法对比了IonPac AS18、IonPac AS11-HC及IonPac AS15 3种类型色谱柱(规格均为250 mm×2 mm),其中IonPac AS15耐受基体能力较强,但灵敏度弱于IonPac AS18及IonPac AS11-HC; IonPac AS11-HC色谱柱中F^-^与乳酸及乙酸分离度弱于IonPac AS18(乳酸及乙酸为常见有机酸,具有潜在污染样品的可能),综合考虑,选择IonPac AS18色谱柱作为分析柱。

### 2.3 方法学考察

#### 2.3.1 线性关系、检出限和定量限

按照1.2节配制混合标准溶液,按照1.4节色谱条件,由低浓度到高浓度依次进样分析,以各离子响应峰面积与质量浓度进行线性回归,以信噪比≥3和10确定仪器的检出限(LOD)和定量限(LOQ)。结果表明,各离子在各自线性范围内具有良好的线性关系,相关系数(*r*)≥0.9994,仪器的检出限和定量限分别为0.05~0.88 μg/L和0.15~2.92 μg/L(见表1)。

**表 1:** 目标离子线性范围、回归方程、相关系数、检出限及定量限

Compound	Regression equation	Linear range/ (μg/L)		r	LOD/ (μg/L)	LOQ/ (μg/L)
F^-^	Y=3.0350X+0.1280	5-	4000	0.9994	0.05	0.15
Cl^-^	Y=0.0850X-0.0020	5-	200	0.9994	0.24	0.79
N	Y=0.0585X+0.0045	5-	200	0.9996	0.40	1.34
Br^-^	Y=0.0369X-0.0127	5-	200	0.9995	0.88	2.92
N	Y=0.0478X-0.0172	5-	200	0.9996	0.66	2.20
S	Y=2.1422X+0.3097	5-	4000	0.9995	0.47	1.55

*Y*: peak area, μS×min; *X*: mass concentration, μg/L.

#### 2.3.2 精密度和稳定性

按1.3节方法制备1.0%碳酸锂溶液,加入适量混合标准溶液,使得Cl^-^、 ${{\mathrm{NO}}_{2}}^{-}$ 、Br^-^、 ${{\mathrm{NO}}_{3}}^{-}$ 质量浓度均为20 μg/L, F^-^及 ${{\mathrm{SO}}_{4}}^{2-}$ 质量浓度均为100 μg/L,以同一天6份样品平行测定峰面积的相对标准偏差(RSD)评价方法的精密度。结果显示,F^-^、Cl^-^、 ${{\mathrm{NO}}_{2}}^{-}$ 、Br^-^、 ${{\mathrm{NO}}_{3}}^{-}$ 和 ${{\mathrm{SO}}_{4}}^{2-}$ 精密度分别为0.09%、0.12%、0.46%、0.33%、0.45%和0.73%,表明该方法重复性良好。

同时将制得的1.0%碳酸锂溶液于0、2、4、8、12、18、24 h间隔进样测定,测得F^-^、Cl^-^、 ${{\mathrm{NO}}_{2}}^{-}$ 、Br^-^、 ${{\mathrm{NO}}_{3}}^{-}$ 和 ${{\mathrm{SO}}_{4}}^{2-}$ 峰面积的RSD值分别为0.15%、0.38%、0.73%、0.49%、0.88%和0.96%,表明碳酸锂样品采用水溶方法24 h内稳定。

#### 2.3.3 回收率

选择已知阴离子含量的碳酸锂进行加标回收试验,精密称取0.5 g碳酸锂,添加阴离子混合标准溶液至样品中,用超纯水超声辅助溶解并定容至50 mL。按照1.3节方法制备样品溶液,计算回收率。结果见表2,碳酸锂样品中各离子的加标回收率为93.3%~99.3%, RSD为0.97%~3.45%(*n*=6),表明本方法的准确性及重复性良好。

**表 2:** 碳酸锂样品中阴离子在3个水平下的回收率(*n*=6)

Compound	Background/ (mg/kg)	Added/ (mg/kg)	Found/ (mg/kg)	Recovery/ %	RSD/ %
F^-^	89.70	20.0	108.40	93.5	0.97
		100.0	186.20	96.5	1.05
		200.0	285.50	97.9	0.99
Cl^-^	0.55	0.3	0.83	93.3	2.58
		0.5	1.04	98.0	1.34
		1.0	1.52	97.0	1.29
N	0.58	0.3	0.86	93.3	3.45
		0.5	1.06	96.0	2.39
		1.0	1.55	97.0	1.87
Br^-^	-	0.3	0.29	96.7	2.68
		0.5	0.48	96.0	2.05
		1.0	0.96	96.0	1.23
N	0.78	0.3	1.06	93.3	3.34
		0.5	1.25	94.0	2.66
		1.0	1.76	98.0	2.73
S	173.20	20.0	192.70	97.5	1.32
		100.0	271.80	98.6	1.28
		200.0	371.80	99.3	1.26

-: not detected.

### 2.4 样品分析

按1.4节色谱条件对标准溶液和样品溶液进行分析,色谱图见图2,样品1中各离子含量见表2,样品2中各离子含量如下:F^-^为88.92 mg/kg, Cl^-^为0.63 mg/kg, ${{\mathrm{NO}}_{2}}^{-}$ 为0.52 mg/kg, Br^-^为未检出, ${{\mathrm{NO}}_{3}}^{-}$ 为0.97 mg/kg, ${{\mathrm{SO}}_{4}}^{2-}$ 为185.70 mg/kg。

**图 2:**
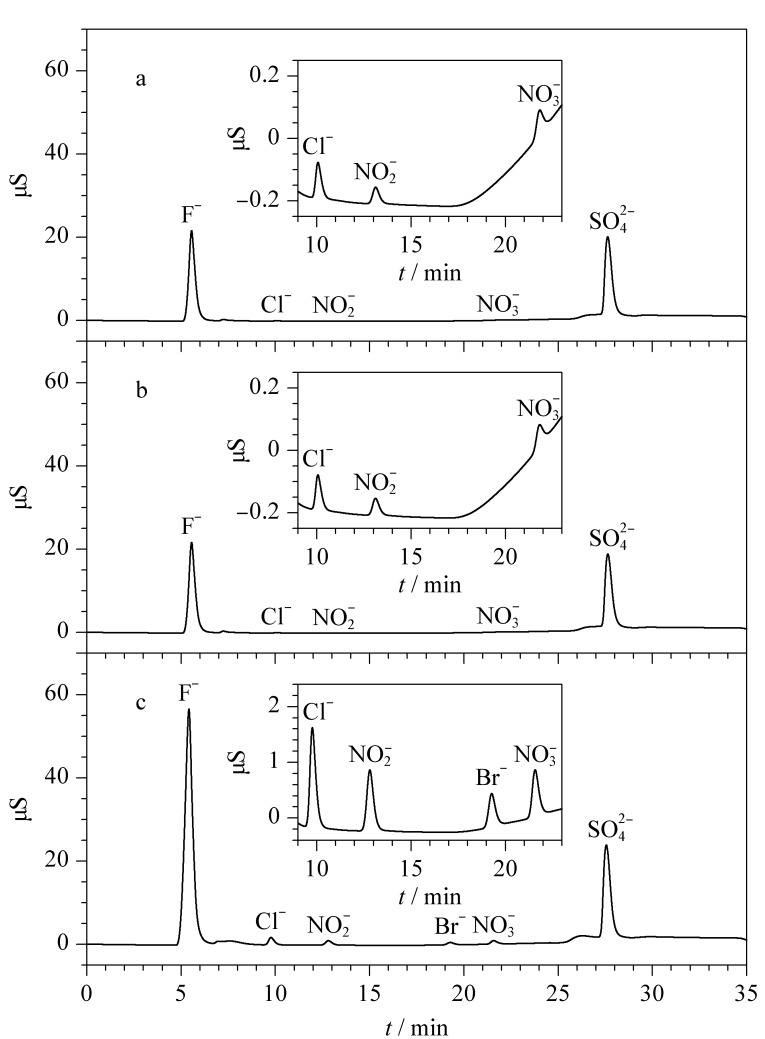
(a)样品1、(b)样品2和(c)标准溶液的色谱图

### 2.5 与已报道方法的比较

本方法各离子的方法定量限根据线性方程最低点计算得到(见表3),可以看出,本方法的方法定量限明显低于文献报道数据,适用于痕量离子的检测。此外,本方法的优势主要如下:超纯水溶解样品,无杂质阴离子引入;连续在线基体去除,可兼容大体积进样,兼容痕量离子检测。

**表 3:** 方法定量限对比

Compound	This method	References				
		[7]	[8]	[9]	[10]	[11]
F^-^	0.5	-	-	-	-	-
Cl^-^	0.5	200.0	100.0	10.0	5.0	-
N	0.5	-	-	-	-	-
Br^-^	0.5	-	-	-	-	-
N	0.5	-	-	-	-	-
S	0.5	200.0	100.0	10.0	5.0	2.7

## 3 结论

本方法使用抑制器及CRD 200在线去除碳酸锂基体,富集后采用离子色谱进行分析,方法检出限低,适用于低含量离子分析,可为高端质量碳酸锂生产提供参考。
